# The Role of Family Functioning and Socioeconomic Context in Multisite and Chronic Musculoskeletal Pain in Adolescents: Generation XXI Cohort Study

**DOI:** 10.3389/ijph.2025.1608929

**Published:** 2025-11-12

**Authors:** Nare Navasardyan, Sonia Bernardes, Ana Henriques, Cláudia F. Oliveira-Gomes, Catarina Pires, Makram Talih, Raquel Lucas

**Affiliations:** 1 EPIUnit ITR, Instituto de Saúde Pública da Universidade do Porto, Universidade do Porto, Porto, Portugal; 2 Department of Social and Organizational Psychology, Centro de Investigação e Intervenção Social (CIS), Iscte – Lisbon University Institute Edifício I, Universidade de Lisboa, Lisbon, Portugal; 3 ANDAI, Associação Nacional de Doentes com Artrites e Reumatismos da Infância, Lisbon, Portugal

**Keywords:** family functioning, chronic musculoskeletal pain, prospective study, socioeconomic factors, adolescence

## Abstract

**Objective:**

We examined whether family functioning relates to multisite and chronic musculoskeletal pain in adolescents, a key etiological stage for chronic pain, considering socioeconomic and childhood adversity factors (ACEs).

**Methods:**

Data from 1,473 participants were analyzed using the Luebeck Pain Screening Questionnaire at 18 years. Multisite pain was defined as pain in ≥2 sites; chronic musculoskeletal pain as pain in any musculoskeletal site lasting >3 months. Family functioning was assessed via the Brief Family Relationship Scale and categorized as poor, fair, or good. Socioeconomic indicators were collected at baseline, and ACEs at age 13.

**Results:**

The prevalence of multisite pain was 43%, and chronic pain was 23%. Logistic regression analyses showed that good family functioning was associated with lower odds of multisite pain (OR 0.49; 95% CI 0.37, 0.65) and chronic musculoskeletal pain (OR 0.62; 95% CI 0.45, 0.86). Socioeconomic indicators had limited effects, though higher maternal occupation was linked to greater multisite pain (OR 1.38; 95% CI 1.02, 1.87). Stratified analyses revealed no significant interactions.

**Conclusion:**

Good family functioning was associated with a lower risk of adolescent pain across socioeconomic contexts.

## Introduction

Chronic musculoskeletal and multisite pain are prevalent health concerns that can significantly affect children and adolescents, potentially leading to long-term disability and a reduced quality of life [1–3]. Research highlights that chronic pain is shaped by a complex interaction of biological, psychological, and social factors, as outlined by the biopsychosocial model of pain [4–6]. Social influences affect how pain is reported and communicated, emphasizing the importance of considering the broader social context in pain research [5].

Among psychosocial factors, families play a key role in shaping children’s pain experiences [7, 8]. Parental behaviors such as modeling, reinforcement, and support can significantly influence pain outcomes, as highlighted in the integrative framework proposed by Palermo and Chambers [9]. Their model emphasizes the interplay between individual parental behaviors, parent–child interactions, and broader family dynamics as a key influence on pediatric chronic pain. For instance, children with supportive and cohesive family environments tend to exhibit better pain management and reduced disability, whereas children from high-conflict families can show worse chronic pain outcomes [10, 11]. However, the relationship between family functioning and pain outcomes may not be uniform across all children, as socioeconomic status (SES), such as household income, parental educational background, and employment status, can interact with family functioning to shape pain experiences [12]. For instance, children from low SES backgrounds with strained family environments may experience more severe pain, not just due to limited resources but also because of compounded psychological and social factors. In contrast, strong family relationships can buffer the negative effects of low SES, enhancing coping skills and resilience [13, 14]. Besides, adverse childhood experiences (ACEs), such as abuse, neglect, and household dysfunction, are individual events and experiences that, within the broader context of socioeconomic challenges, shape childhood environments and long-term outcomes, with SES representing the structural aspect of these challenges [15, 16].

Despite increasing evidence linking family functioning to pain experiences, little is known about how these associations vary across the socioeconomic gradient in youth, a stage of life characterized by significant physical, emotional, and social changes. Most research on family relationships and pain has been conducted in small clinical or treatment-seeking samples, which limits generalizability. Our study addresses this gap by using a large, population-based birth cohort with prospective data and validated measures of family functioning, providing a stronger basis for understanding how these dynamics affect pain in the general population. Previous research has shown that adolescents who report persistent pain are at increased risk of developing chronic pain in adulthood [2]. Importantly, late adolescence represents a critical developmental stage, as it marks the transition into adulthood, a period when musculoskeletal pain may consolidate into more persistent patterns, making it a particularly relevant age to investigate the influence of family functioning and socioeconomic context on pain outcomes.

Thus, we aimed to examine the relationship between family functioning and multisite and chronic musculoskeletal pain at age 18 within a population-based birth cohort, exploring the interaction with socioeconomic indicators and ACEs.

## Methods

### Study Design

We analyzed data from participants from the Generation XXI (G21) population-based birth cohort from Portugal. G21 recruitment took place between April 2005 and August 2006 at all five public maternity units in the metropolitan area of Porto, Portugal [17]. Overall, 8,647 children were included in the cohort at baseline and, subsequently, followed up at ages 4 (2009–2011; 86% participation), 7 (2012–2014; 80% participation), 10 (2015–2017; 74% participation), 13 (2018–2020; 54% participation) and 18 (2022–2025).

Each wave included physical examinations of the participants, comprising anthropometric measurements and blood samples, alongside face-to-face structured interviews conducted by trained interviewers with both the children/youth and their caregivers. These interviews collected clinical, social, and behavioral characteristics such as common diseases and symptoms, school-related issues, and adversity experiences.

### Ethical Considerations

The G21 birth-cohort study complies with the Helsinki Declaration for medical research and current national legislation and was approved by the Ethics Committee of Hospital São João/University of Porto Medical School until the 10-year follow-up wave. This study was approved by the ethics committees of the Institute de Saúde Pública da Universidade do Porto (ISPUP) (Ref CE21199) and Hospital Garcia de Orta (Ref 12/2022). Informed consent was obtained from all adolescents aged 18 and older, and from both caregivers and adolescents when participants were under 18.

### Study Sample and Data Collection

**FIGURE 1:**
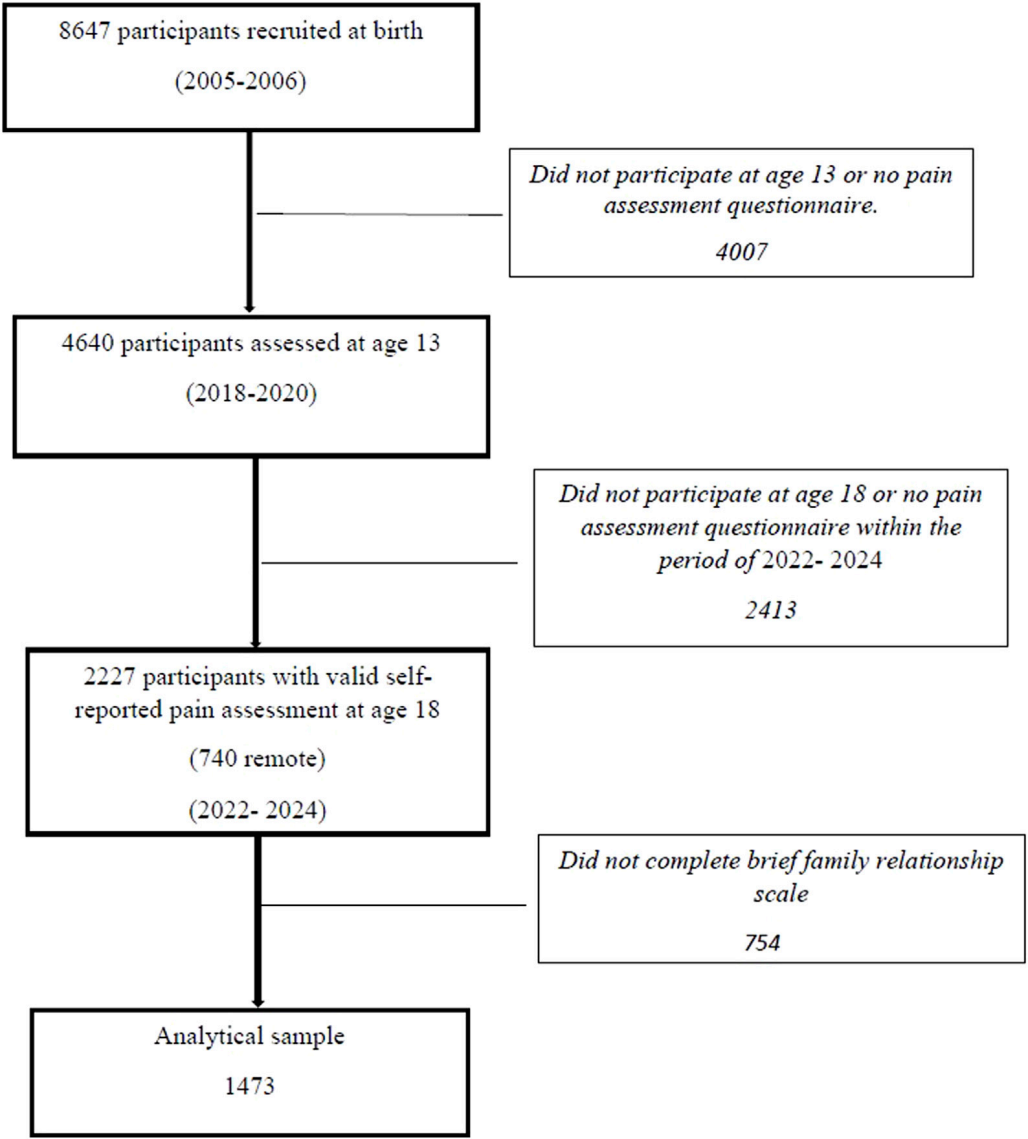
Participants’ flowchart. Generation XXI birth cohort study, Portugal, 2005/6 ‐ 2025.

For this study, we used pain data collected at age 18, a follow-up wave that started remotely in June 2022 through a dedicated mobile application called “SEPIA,” developed in collaboration with the Institute of Systems and Computer Engineering, Technology and Science (INESC TEC) of the University of Porto. The development and implementation of SEPIA involved patient partners, who contributed to the study design, conduct, and interpretation of results, ensuring that the research addressed relevant needs and experiences. The application was intended to collect data about prior pain experiences from each adolescent and one caregiver (92% mothers, who were identified as primary caregivers since recruitment). Caregivers in the cohort who had provided their email addresses were emailed by the research team (5,756 families), and those who agreed to participate were sent a set of links and credentials to access the application (link: https://ispup.up.pt/sepia/). A total of 740 participants provided data remotely through the SEPIA mobile application. Besides pain data, other information, such as data on family functioning, was collected via the application. This collection was complemented with data from the ongoing G21 wave of in-person evaluations, including participants who finished the pain-related assessment by September 2024, using the same application and tools. In this study, we included participants who responded to the Lübeck Pain Screening Questionnaire and the Brief Family Relationship Scale at age 18, from June 2022 to September 2024, using remote and face-to-face modalities, resulting in a final sample of 1,473 (Figure 1).

### Study Variables

#### Multisite Pain and Chronic Musculoskeletal Pain

The Luebeck pain screening questionnaire (LPQ) was developed for epidemiological investigations of pain characteristics among children and adolescents [18]. It has been administered to the cohort since age 7 to evaluate the prevalence, consequences, and self-perceived triggers of pain, as well as its impact on daily living. Additionally, participants are asked if they live with any family members (e.g., parents, grandparents, siblings, others) who have recurrent or chronic pain. The first question of the LPQ asked about any occurrence of pain within the 3 months before the interview, with a negative response bypassing further questions. If the response was affirmative, the participants were asked to describe their experience (self-reports at ages 13 and 18), explicitly identifying pain site(s) and the principal (most bothersome) pain site. For the principal site, LPQ collected information on duration, which was recorded as less than 3 months, 3–12 months, and more than 12 months. As the primary outcomes, we assessed multisite pain and chronic musculoskeletal pain. Multisite pain was defined as pain in two or more specific sites out of twenty-six possible locations, including the head, abdomen, pelvis, lower/mid back, neck/shoulders, upper and lower limbs, and hips, within the past 3 months. Chronic musculoskeletal pain was defined as pain in any of the principal musculoskeletal sites (lower/mid back, neck/shoulders, upper and lower limbs, or hips) that persisted for more than 3 months.

#### Family Functioning

Family functioning was assessed using a Portuguese version of the Brief Family Relationship Scale (BFRS). The BFRS tool aims to assess a young individual’s perception of the quality of family relationship functioning [19, 20]. It has 16 items, each item having a binary (true-false) response, with three dimensions measuring:

- cohesion, i.e., degree of emotional bonding, support, and commitment family members have towards each other (7 items),
- conflict, i.e., level of openly expressed aggression, anger and conflict among family members (6 items),
- expressiveness, i.e., level of open communication and emotional sharing within the family (3 items).

Based on our exploratory factor analysis and the nuanced meaning of the word “discussion” (“discussão”) in Portuguese, we decided to exclude Item 15 from the questionnaire (“In our family, we begin discussions easily”), resulting in a final version with 15 items (Supplementary Table S1).

Consistent with prior research using the Brief Family Relationship Scale, we computed an overall family functioning score [21]. The final family functioning score ranged from 0 to 15, with cohesion ranging from 0 to 7, conflict from 0 to 6, and expressiveness from 0 to 2. For the final score, the conflict subscale was reversed so that a higher overall score indicates better family functioning. The internal consistency of the scale, as measured by Cronbach’s alpha, is 0.8256.

#### Socioeconomic Variables

Baseline data on monthly household income, maternal education, and parental occupation were analyzed. The income included wages and other sources (e.g., financial aid, rent, allowances) and was categorized as low (≤EUR 1,000), intermediate (EUR 1,001–2,000), and high (>EUR 2,000), based on Portugal’s minimum wage [22]. Maternal education was categorized per the International Standard Classification of Education 2011: low (≤9 years), intermediate (10–12 years), and high (>12 years) [23]. Parental occupations were categorized into major professional groups based on the National Classification of Occupations and further collapsed into three levels: low (blue-collar professions such as farmers, craftsmen, machine operators, assembly workers, and skilled or unskilled laborers); intermediate (lower white-collar roles including administrative staff, service workers, and sales personnel); and high (upper white-collar positions such as executive civil servants, industrial managers, scientists, middle managers, and technicians) [22, 24].

#### Adverse Childhood Experiences

ACEs were assessed at age 13 using a 15-question survey based on the original ACEs study [16, 25]. Adolescents completed it privately with a trained interviewer available for assistance. ACE exposure was determined by summing occurrences of each adversity and was scored binary (0/1). Total adversity was grouped into three categories: 0–3 ACEs, 4–5 ACEs, and ≥6 ACEs, based on their association with pain experiences [26]. Prevalence data for our sample can be found in Supplementary Table S2.

### Statistical Analysis

Group comparisons were performed using chi-square (χ^2^) tests for categorical variables.

Binary logistic regression models were built to examine the associations of family functioning, SES indicators, and ACEs with pain outcomes (multisite and chronic musculoskeletal pain). Crude odds ratios (OR) with their corresponding 95% confidence intervals (95% CI) were reported to describe associations.

To enhance the visualization of our results, based on data distribution, we categorized the overall family functioning score (mean = 11.6, median = 12) into three groups: poor (0–9), fair (10–12), and good (13–15). Stratified analyses using chi-square (χ^2^) tests were conducted to explore subgroup differences in the prevalence of pain outcomes (multisite and chronic musculoskeletal pain) by family functioning (relationship categories 0–9, 10–12, 13–15) across SES levels and by number of ACEs.

## Results

Among the 1,473 participants (52.5% female), more than half belonged to middle or high-income households and were born to mothers with intermediate or higher educational levels. Also, more than half reported experiencing pain in the past 3 months, 43% reported multisite pain, and approximately one-fourth reported chronic musculoskeletal pain. Almost half of the participants indicated living with a family member who has chronic or persistent pain, of whom more than two-thirds reported that it was their mother experiencing pain (Table 1).

**TABLE 1:** Sample characteristics (n = 1,473). Generation XXI birth cohort study, Portugal, 2005/6 ‐ 2025.

Characteristics	n (%)
Sex Male Female	699 (47.5) 774 (52.5)
Monthly household income <1,000€ 1,000–2000€ >2000€ Missing	412 (31.8) 646 (49.9) 237 (18.3) *178*
Maternal education <10 10–12 >12 Missing	448 (31.2) 465 (32.4) 524 (36.4) *36*
Maternal occupation Blue collar Lower white collar Upper white collar Missing	262 (18.8) 622 (44.6) 511 (36.6) *78*
Paternal occupation Blue collar Lower white collar Upper white collar Missing	56 (34.7) 241 (18.3) 617 (47.0) *159*
Adverse childhood experiences 0–3 4–5 6+ Missing	704 (53.3) 330 (25.0) 286 (21.7) *153*
Any pain (previous 3 months) Yes No	750 (50.9) 723 (49.1)
Multisite pain (2 or more) Yes No	629 (42.7) 844 (57.3)
Chronic musculoskeletal pain Yes No Missing	335 (22.8) 1,132 (77.2) *6*
Family member with pain Yes No	648 (44.0) 825 (56.0)
Mother with pain Yes No	464 (31.5) 1,009 (68.5)
Father with pain Yes No	204 (13.9) 1,269 (86.1)
Siblings with pain Yes No	35 (2.4) 1,438 (97.6)
Other family members with pain Yes No	65 (4.4) 1,408 (95.6)

Italic values indicate missing data.

We observed sex differences in pain reports, with males having significantly lower odds of reporting multisite pain (OR 0.40; 95% CI 0.32, 0.49) and chronic musculoskeletal pain (OR 0.74; 95% CI 0.58, 0.95) compared to females. Table 2 presents the crude association between socioeconomic indicators, ACEs, family functioning, and multisite and chronic musculoskeletal pain. Among all socioeconomic factors tested, only maternal occupation showed a statistically significant association with multisite pain (OR for upper white-collar vs. blue-collar: 1.38; 95% CI 1.02, 1.87), whereas no statistically significant associations were found between any of the socioeconomic indicators and chronic musculoskeletal pain.

**TABLE 2:** Crude associations between socioeconomic indicators, ACEs, family functioning, and pain outcomes. Generation XXI birth cohort study, Portugal, 2005/6 ‐ 2025.

Variables	Unadjusted model OR [95%CI]	
	Multisite pain (2 or more sites)	Chronic musculoskeletal pain
Sex Female Male	1^a^ **0.40 [0.32, 0.49]**	1^a^ **0.74 [0.58, 0.95]**
Monthly household income <1,000€ 1,000–2000€ >2000€	1^a^ 1.15 [0.89, 1.48] 1.33 [0.96, 1.84]	1^a^ 1.13 [0.84, 1.53] 1.07 [0.73, 1.58]
Maternal education <10 10–12 >12	1^a^ 1.24 [0.95, 1.31] 1.20 [0.93, 1.56]	1^a^ 1.12 [0.82, 1.53] 1.23 [0.92, 1.67]
Maternal occupation Blue collar Lower white collar Upper white collar	1^a^ 1.33 [0.99, 1.80] **1.38 [1.02, 1.87]**	1^a^ 1.34 [0.94, 1.93] 1.39 [0.96, 2.02]
Paternal occupation Blue collar Lower white collar Upper white collar	1^a^ 0.89 [0.65, 1.23] 1.11 [0.87, 1.41]	1^a^ 0.97 [0.66, 1.42] 1.15 [0.87, 1.54]
ACEs 0–3 4–5 6+	1^a^ 1.12 [0.86, 1.45] 1.27 [0.97, 1.68]	1^a^ 0.89 [0.65, 1.23] 1.12 [0.82, 1.55]
Cohesion (0–7)	**0.83 [0.77, 0.89]**	**0.88 [0.81, 0.95]**
Conflict (0–6)	**1.16 [1.09, 1.23]**	**1.11 [1.03, 1.19]**
Expressiveness (0–2)	**0.83 [0.69, 0.99]**	0.87 [0.71, 1.08]
Family functioning score (total 0–15)	**0.91 [0.88, 0.94]**	**0.93 [0.90, 0.97]**
Family functioning Poor (0–9) Fair (10–12) Good (13–15)	1^a^ **0.71 [0.53, 0.96]** **0.49 [0.37, 0.65]**	1^a^ 0.92 [0.66, 1.28] **0.62 [0.45, 0.86]**

^a^ Reference category for each variable is indicated by OR = 1. Bold values indicate statistically significant results.

Participants who reported six or more ACEs had a slightly increased likelihood of reporting multisite pain (OR 1.27; 95% CI: 0.97, 1.68), but this association was not statistically significant. No other statistically significant associations were found between ACEs and reports of multisite or chronic musculoskeletal pain.

Having good family functioning significantly decreased the odds of reporting multisite pain and chronic musculoskeletal pain (OR 0.49; 95% CI 0.37, 0.65; OR 0.62; 95% CI 0.45, 0.86, respectively). Higher family cohesion was associated with lower odds of pain outcomes (for multisite: OR 0.83; 95% CI 0.77, 0.89; for chronic musculoskeletal: OR 0.88; 95% CI 0.81, 0.95), whereas higher levels of family conflict increased the odds of both multisite and chronic musculoskeletal pain (OR 1.16; 95% CI 1.09, 1.23; OR 1.11; 95% CI 1.03, 1.19 respectively).

**FIGURE 2:**
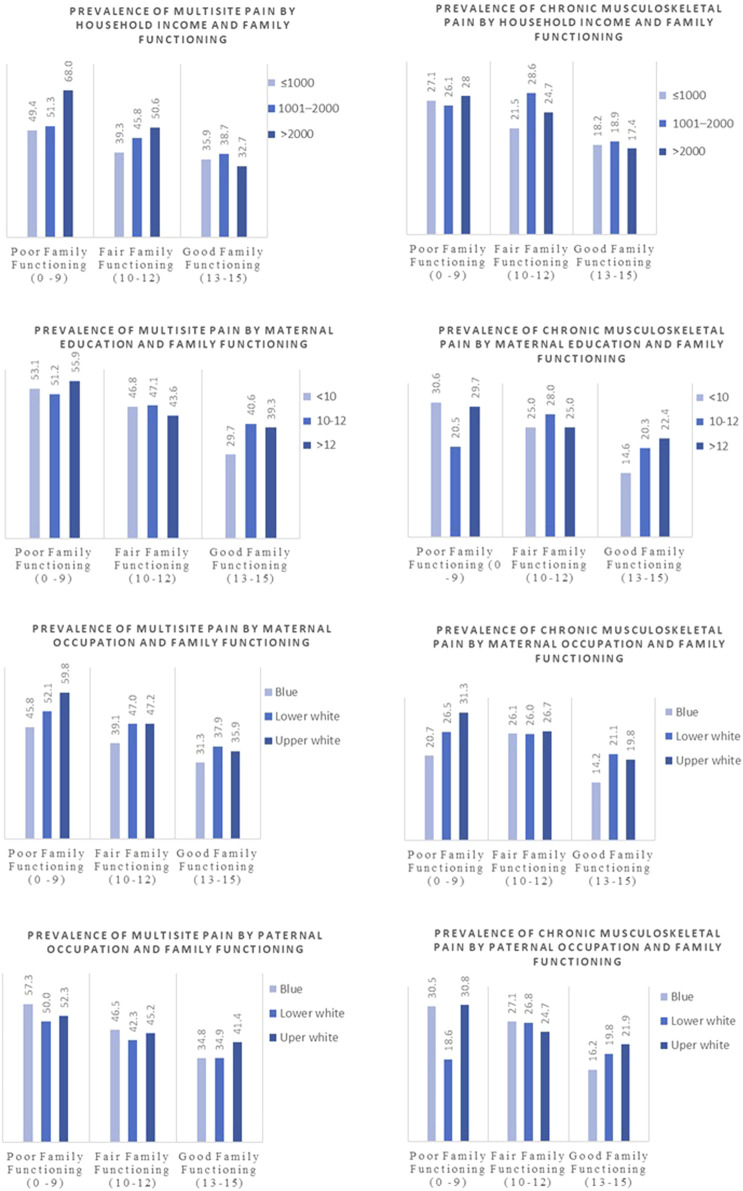
Prevalence of multisite and chronic musculoskeletal pain (%) by socioeconomic indicators within categories of family functioning. Generation XXI birth cohort study, Portugal, 2005/6 ‐ 2025.

**FIGURE 3:**
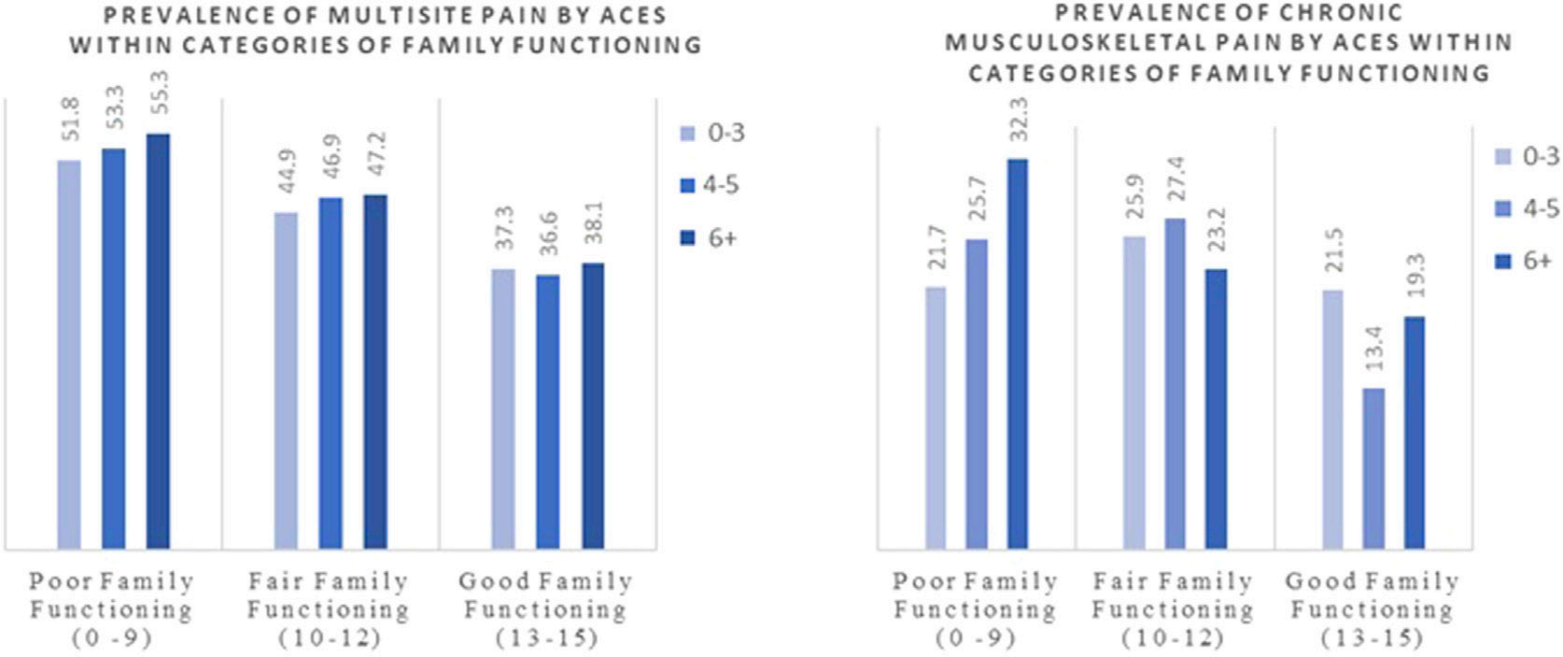
Prevalence of multisite and chronic musculoskeletal pain (%) by ACEs within categories of family functioning. Generation XXI birth cohort study, Portugal, 2005/6 ‐ 2025.

Stratified analyses revealed no significant interactions between family functioning and socioeconomic indicators or ACEs on pain outcomes. Nonetheless, adolescents from families with good functioning consistently reported a lower prevalence of chronic musculoskeletal and multisite pain across all levels of socioeconomic indicators and adverse childhood experiences (Supplementary Tables S3, S4). Only within the ‘good family functioning’ subgroup, the association between family functioning and pain outcomes was less pronounced for participants with mothers who had lower maternal education (Figures 2, 3). Specifically, participants with lower maternal education reported a lower prevalence of multisite pain (29.7%) compared to those with intermediate or higher maternal education (40.6% and 39.3%, respectively; p = 0.030 across the three categories). A similar association was observed for chronic musculoskeletal pain in the same subgroup, showing borderline significance (14.6%, 20.3%, and 22.4%, respectively; p = 0.091) (Supplementary Table S4).

## Discussion

In this study, we examined the association between family functioning and pain outcomes according to different socioeconomic indicators and childhood adversities. Our results underscore that adolescents from families with poor family functioning, characterized by low cohesion, expressiveness, and high conflict, were more likely to report multisite pain or chronic musculoskeletal pain, across levels of socioeconomic factors and childhood adversities. Supportive and cohesive family environments were associated with lower odds of multisite and chronic musculoskeletal pain. In contrast, higher family conflict was associated with higher odds. These findings highlight the relevance of family relationships to adolescent pain experiences, consistent with the biopsychosocial model’s emphasis on the role of immediate social environments in pain [11]. Interestingly, socioeconomic factors and adverse childhood experiences, while important in shaping broader health outcomes, played a less prominent role in modifying these relationships at the age of 18.

Our results are similar to findings from a study in the United States, which showed that greater pain was associated with increased functional disability in children aged 7–17 years with recurrent migraines from disruptive family environments, but not in those from more adaptive family environments [10]. Similarly, a study by Palermo et al. demonstrated that adolescents aged 11–16 with recurrent headaches and less healthy family functioning reported more pain and disability [27]. The stress and emotional dysregulation caused by family conflict can lead to heightened central sensitization, exacerbating pain perception, which underlines the importance of addressing family conflict in pain management interventions [28]. Evidence from juvenile rheumatic diseases further underscores the bidirectional nature of this relationship: children reporting greater symptom severity, such as more days of pain and fatigue, tended to exhibit poorer attitudes toward their illness and experienced lower levels of family functioning [29].

Our data showed that females were significantly more likely to report multisite and chronic musculoskeletal pain than males, a pattern consistently found in previous studies. For example, a Danish cross-sectional study reported that girls aged 12–19 were nearly twice as likely as boys to report multisite pain [3]. This higher prevalence may reflect a mix of physiological and psychosocial factors, though it remains unclear whether it is primarily biological or influenced by broader factors not assessed in this study [30].

Despite prior research linking socioeconomic status to pain prevalence, our study found that SES indicators, including household income and parental occupation, had limited influence on the relationship between family functioning and pain at age 18 [31]. A systematic review by Sara King and colleagues found no definitive association between socioeconomic status and pain prevalence in children and adolescents [32]. One study within the review reported a higher prevalence of pain in low SES groups, particularly headaches, while others found no association between pain and SES. Another study from Nordic countries showed that children aged 7–17 years from low-educated or low-income families had a slightly higher prevalence of pain in comparison to those of high status [33]. Although this was not the main focus of our paper, the socioeconomic gradient in pain prevalence appears to be more pronounced in adult populations. A pan-European study showed that pain was more prevalent among adults with lower socioeconomic status, with the greatest disparities observed for hand and arm pain and the smallest for back and neck pain [34]. This well-defined gradient in adults may result from cumulative exposure to socioeconomic stressors and occupational hazards over time. In contrast, this relationship is less evident in children and adolescents, possibly due to stronger comparative effects of growth, development, and family functioning on pain experiences. Interestingly, while looking at the prevalence of multisite pain by maternal education and family functioning, we found that among adolescents reporting good family relationships, those whose mothers had higher education levels reported greater pain prevalence compared to those with mothers of low or intermediate education. This could be explained by the higher health literacy often associated with maternal education, which may lead to greater awareness and reporting of pain [35]. This could be explained by the higher health literacy often associated with maternal education, which may lead to greater awareness and reporting of pain [35]. For instance, mothers with higher education may be more attentive to subtle health complaints or more likely to encourage their children to report symptoms, potentially inflating the observed prevalence in this subgroup. In addition, higher educational attainment may be linked to increased awareness of health risks and greater access to health information, amplifying pain reporting without necessarily reflecting higher true morbidity. Alternatively, the pressures of high-achieving households may contribute to increased stress and, consequently, higher pain prevalence even in supportive families, underscoring the complex interplay between family functioning, SES, and adolescent pain experiences [36]. These explanations remain hypotheses, and future research should investigate the underlying psychosocial and contextual mechanisms linking maternal education and adolescent pain, including household stress, parenting practices, or health-awareness behaviors, to better understand these associations.

While prior studies have found a clear association between a higher number of adverse childhood experiences and increased pain prevalence, our data showed some weak evidence supporting this [16, 37]. We examined ACE reports from age 13 to pain reports at age 18, whereas previous research in this cohort found that ACEs during the first decade of life were associated with pain reports at age 13, but it specifically focused on multisite, high-intensity pain [16]. Furthermore, the sample size in the previous study was larger, which could have influenced the results. The trajectory of pain development and its mediators, such as coping strategies, social support, and mental health, may differ significantly between adolescence and earlier childhood, which could further explain the differences in our findings.

Our findings contribute to a growing body of literature on the interplay between family functioning and adolescent pain while revealing nuances specific to the Portuguese context. In countries like Portugal where the collectivism level in terms of the role of the family in society and child-rearing is high, family functioning may often play an even more pronounced role in health outcomes that could contrast with findings from more individualistic cultures, where the impact of family functioning may be moderated by peer and community influences [38, 39]. These cultural differences may limit direct comparisons or generalizations to settings with different population distributions and family structures, particularly in countries with more diverse or less cohesive family dynamics.

The strengths of this study lie in its large, population-based cohort, which, to our knowledge, is the first to investigate the relationship between family functioning and pain in youth across the social gradient, an area that remains underexplored. Unlike much prior research based on clinical pain samples often involving treatment-seeking individuals, our use of a population-based sample includes adolescents who may not be actively seeking care or experiencing significant functional impairment. This approach allows us to investigate potential risk and protective factors before pain reaches clinical thresholds. At the same time, we consider this pain to be meaningful, as evidenced by recent findings from the same cohort showing that multisite or chronic musculoskeletal pain in late adolescence is associated with lower quality of life [40]. This distinction enhances the generalizability of our findings and supports their relevance for population-level prevention strategies. However, there are several limitations. While our study primarily focused on whether family functioning affects pain prevalence, our results can also be interpreted in the opposite direction, as evidence that pain can influence family functioning, reinforcing the bidirectional nature of this relationship (Supplementary Table S5). Additionally, using data from a population-based cohort with varying levels of attrition may have led to sociodemographic differences between participants and non-participants. However, prior analysis within this cohort found no significant associations between pain characteristics and sociodemographic factors, except for maternal education [41]. Furthermore, reliance on self-reported measures for family functioning could introduce recall or social desirability biases, potentially impacting the accuracy of the reported data. Finally, the generalizability of our findings may be limited to populations with similar sociocultural contexts, healthcare systems, and family structures. Residual confounding from unmeasured psychological distress at the time of reporting, personality traits, and a more comprehensive assessment of different family members’ functioning may also influence the observed associations. Additionally, several aspects of the physical environment, such as living conditions, were not assessed.

This study highlights the central role of family functioning in shaping adolescent pain experiences across the socioeconomic gradient, emphasizing that good family environments can have protective effects, whereas high family conflict may exacerbate pain. Although socioeconomic factors and adverse childhood experiences are known to influence broader health outcomes, their isolated contribution to the etiology of multisite or chronic musculoskeletal pain appears limited in adolescence. This suggests that universal interventions aimed at fostering family cohesion and conflict resolution could benefit adolescents across all socioeconomic backgrounds. A deeper understanding of these relationships, particularly within diverse cultural and socioeconomic contexts, is needed to refine our understanding and inform future approaches.

## Data Availability

The data from Generation XXI are not publicly available due to privacy or ethical restrictions. The data can be made available for research proposals on request to the Generation XXI Executive Committee (generationxxi@ispup.up.pt).
