# Supplementary material for: The Role of Family Functioning and Socioeconomic Context in Multisite and Chronic Musculoskeletal Pain in Adolescents: Generation XXI Cohort Study
Source: Int J Public Health. 2025 Nov 12;70:1608929. doi: 10.3389/ijph.2025.1608929 (PMC12646965; doi:10.3389/ijph.2025.1608929)
Supplement: Supplementary file 1 [file Supplementaryfile1.docx]

International Journal of Public Health

**The Role of Family Functioning and Socioeconomic Context in Multisite and Chronic Musculoskeletal Pain in Adolescents: Generation XXI Cohort Study**

Supplementary Table 1. Brief Family Relationship Scale (n=1473)

| **Items** | True - 1 | False - 0 |
| --- | --- | --- |
| 1. Na nossa família nós ajudamo-nos e apoiamo-nos uns aos outros.   In our family we really help and support each other. (**cohesion)** | 1399 (95.0%) | 74 (5.0%) |
| 1. Na nossa família discutimos muito.   In our family we argue a lot. *(conflict)* | 503 (34.2%) | 970 (65.8%) |
| 1. Na nossa família passamos muito tempo a fazer coisas juntos em casa.   In our family we spend a lot of time doing things together at home. **(cohesion)** | 759 (51.5) | 714 (48.5%) |
| 1. Na nossa família podemos falar abertamente em casa.   In our family we can talk openly in our home. (expressiveness) | 1312 (89.1%) | 161 (10.9%) |
| 1. Na nossa família zangamo-nos muito uns com os outros.   In our family we are really mad at each other a lot. (*conflict)* | 394 (26.8%) | 1079(73.2%) |
| 1. Na nossa família empenhamo-nos muito nas tarefas caseiras.   In our family we work hard at what we do in our home. **(cohesion)** | 1122 (76.2%) | 351 (23.8%) |
| 1. Na nossa família há um sentimento de união.   In our family there is a feeling of togetherness. (**cohesion)** | 1318 (89.5%) | 155(10.5%) |
| 1. Na nossa família, por vezes, falamos sobre os nossos problemas pessoais.   In our family we sometimes tell each other about our personal problems. (expressiveness) | 1250 (85.0%) | 223 (15.0%) |
| 1. Na nossa família perdemos a paciência muitas vezes.   In our family we lose our tempers a lot. *(conflict)* | 789 (53.6%) | 684 (46.4%) |
| 1. Na nossa família estamos, muitas vezes a deitar-nos uns aos outros abaixo.   In our family we often put down each other. *(conflict)* | 160 (10.9%) | 1313 (89.1%) |
| 1. Os membros da minha família apoiam-se.   My family members really support each other. (**cohesion)** | 1402 (95.2%) | 71 (4.8%) |
| 1. Os membros da minha família são, por vezes, violentos.   My family members sometimes are violent. *(conflict)* | 120 (8.1%) | 1353 (91.9%) |
| 1. Sinto orgulho por fazer parte da nossa família.   I am proud to be a part of our family. **(cohesion)** | 1406 (95.5%) | 67 (4.5%) |
| 1. Na nossa família damo-nos muito bem uns com os outros.   In our family we really get along well with each other. **(cohesion)** | 1313 (89.1%) | 160 (10.9%) |
| 1. Na nossa família começamos discussões com facilidade. *   In our family we begin discussions easily. (expressiveness) | 554 (37.6%) | 919 (62.4%) |
| 1. Na nossa família levantamos a voz quando estamos zangados.   In our family we raise our voice when we are mad. *(conflict)* | 1006 (68.3%) | 467 (31.7%) |

*Item 15 was excluded based on exploratory factor analysis (EFA), which showed a negative loading on the expressiveness factor. Confirmatory factor analysis (CFA) demonstrated improved model fit without this item (RMSEA = 0.068, CFI = 0.904, SRMR = 0.061) compared to the 16-item version (RMSEA = 0.078, CFI = 0.872, SRMR = 0.074). Linguistic analysis and back-translation revealed that the Portuguese term "discussão" often implies conflict, leading to misinterpretation of the item.

Supplementary Table 2. Prevalence of adverse childhood experiences assessed at age 13 in our sample

| **Adverse childhood experiences, n (%)** |  | |
| --- | --- | --- |
|  | **No** | **Yes** |
| Parents ever separated or divorced | 1083(79.8) | 274 (20.2) |
| Moved from a house, school, or neighborhood | 848(62.4) | 510(37.6) |
| Difficulties in school | 942 (69.9) | 406 (30.1) |
| Witnessing parents arguing or fighting | 734 (54.2) | 620 (45.8) |
| Financial issues in the household | 1060 (78.2) | 296 (21.8) |
| Death of a family member | 729 (53.6) | 630 (46.4) |
| Injury or severe illness of a family member | 802 (59.1) | 556 (40.9) |
| Bullying victimization at school | 1154 (85.2) | 200 (14.8) |
| Household member alcohol abuse or drug addiction | 1344 (98.2) | 25 (1.8) |
| Household member went to prison | 1325 (97.6) | 33 (2.4) |
| Child hospitalization due to a disease or an accident | 1234 (91.0) | 122 (9.0) |
| Household member shout, yelled, or screamed at the child | 610 (44.9) | 748 (55.1) |
| Household member swore, insulted, put down, or humiliated the child | 1233 (90.9) | 124 (9.1) |
| Household member hit, kicked or punched the child | 1242 (91.7) | 113 (8.3) |
| Parents called to school because child did something wrong | 1140 (84.1) | 216 (15.9) |

Supplementary Table 3. Stratified analyses of family functioning and multisite pain reports by socioeconomic indicators and ACEs

|  | | Multisite pain (2 or more sites) | | P value |
| --- | --- | --- | --- | --- |
|  |  | Yes (%) | No (%) |  |
| Poor family functioning (0-9)  *Household income 1*  *Household income 2*  *Household income 3* | Total – 252  85  117  50 | 42(49.4)  60(51.3)  34(68.0) | 43­(50.6)  57(48.7)  16(32.0) | 0.082 |
| Fair family functioning (10-12)  *Household income 1*  *Household income 2*  *Household income 3* | Total – 427  135  203  89 | 53(39.3)  93(45.8)  45(50.6) | 82(60.7)  110(54.2)  44(49.4) | 0.228 |
| Good family functioning (13-15)  *Household income 1*  *Household income 2*  *Household income 3* | Total – 616  192  326  98 | 69(35.9)  126(38.7)  32(32.7) | 123(64.1)  200(61.3)  66(67.3) | 0.531 |
|  | 1295 |  | |  |
| Poor family functioning (0-9)  *Maternal education 1*  *Maternal education 2*  *Maternal education 3* | Total- 284  98  84  102 | 52(53.1)  43(51.2)  57(55.9) | 46(46.9)  41(48.8)  45(44.1) | 0.810 |
| Fair family functioning (10-12)  *Maternal education 1*  *Maternal education 2*  *Maternal education 3* | Total- 469  124  157  188 | 58(46.8)  74(47.1)  82 (43.6) | 66(53.2)  83(52.9)  106(56.4) | 0.773 |
| Good family functioning (13-15)  *Maternal education 1*  *Maternal education 2*  *Maternal education 3* | Total- 684  226  224  234 | 67(29.7)  91(40.6)  92(39.3) | 159(70.3)  133(59.4)  142(60.7) | **0.030** |
|  | 1437 |  |  |  |
| Poor family functioning (0-9)  *Maternal occupation 1*  *Maternal occupation 2*  *Maternal occupation 3* | Total- 277  59  121  97 | 27(45.8)  63(52.1)  58(59.8) | 32(54.2)  58(47.9)  39(40.3) | 0.216 |
| Fair family functioning (10-12)  *Maternal occupation 1*  *Maternal occupation 2*  *Maternal occupation 3* | Total- 449  69  200  180 | 27(39.1)  94(47.0)  85(47.2) | 42(60.9)  106(53.0)  95(52.8) | 0.473 |
| Good family functioning (13-15)  *Maternal occupation 1*  *Maternal occupation 2*  *Maternal occupation 3* | Total- 669  134  301  234 | 42(31.3)  114(37.9)  84(35.9) | 92(68.7)  187(62.1)  150(64.1) | 0.423 |
|  | Total- 1395 |  |  |  |
| Poor family functioning (0-9)    *Paternal occupation 1*  *Paternal occupation 2*  *Paternal occupation 3* | Total – 256  82  44  130 | 47(57.3)  22(50.0)  68(52.3) | 35(42.7)  22(50.0)  62(47.7) | 0.680 |
| Fair family functioning (10-12)  *Paternal occupation 1*  *Paternal occupation 2*  *Paternal occupation 3* | Total – 434  144  71  219 | 67(46.5)  30(42.3)  99(45.2) | 77(53.5)  41(57.7)  120(54.8) | 0.839 |
| Good family functioning (13-15)  *Paternal occupation 1*  *Paternal occupation 2*  *Paternal occupation 3* | Total – 624  230  126  268 | 80(34.8)  44(34.9)  111(41.4) | 150(65.2)  82(65.1)  157(58.6) | 0.243 |
|  | Total- 1314 |  |  |  |
| Poor family functioning (0-9)  *ACEs* 0-3  *ACEs 4-5*  *ACEs 6+* | Total – 252  83  75  94 | 43(51.8)  40 (53.3)  52(55.3) | ­40(48.2)  35 (46.7)  42(44.7) | 0.895 |
| Fair family functioning (10-12)  *ACEs* 0-3  *ACEs 4-5*  *ACEs 6+* | Total – 426  205  113  108 | 92(44.9)  53(46.9)  51(47.2) | 113(55.1)  60(53.1)  57(52.8) | 0.902 |
| Good family functioning (13-15)  *ACEs* 0-3  *ACEs 4-5*  *ACEs 6+* | Total – 642  416  142  84 | 155(37.3)  52(36.6)  32(38.1) | 261(62.7)  90(36.6)  52(61.9) | 0.975 |
|  | Total 1320 |  |  |  |

household income 1(<1000), 2(1001-2000), 3(>2000); maternal education 1(<10), 2(10-12), 3(>12); maternal occupation 1(blue collar), 2(lower white collar), 3(upper white collar); maternal occupation1(blue collar), 2(lower white collar), 3(upper white collar); maternal age 1(13-25), 2(26-35), 3(36-47)

Supplementary Table 4. Stratified analyses of family functioning and chronic musculoskeletal pain reports by socioeconomic indicators and ACEs

|  | | Chronic musculoskeletal pain | | P value |
| --- | --- | --- | --- | --- |
|  |  | Yes (%) | No (%) |  |
| Poor family functioning (0-9)  *Household income 1*  *Household income 2*  *Household income 3* | Total -250  85  115  50 | 23(27.1)  30(26.1)  14(28.0) | 62(72.9)  85(73.9)  36(72.0) | 0.966 |
| Fair family functioning (10-12)  *Household income 1*  *Household income 2*  *Household income 3* | Total - 427  135  203  89 | 29(21.5)  58(28.6)  22(24.7) | 106(78.5)  145(71.4)  67(75.3) | 0.336 |
| Good family functioning (13-15)  *Household income 1*  *Household income 2*  *Household income 3* | Total- 612  192  322  98 | 35(18.2)  61(18.9)  17(17.4) | 157(81.8)  261(81.1)  81(82.6) | 0.934 |
|  | Total- 1289 |  | |  |
| Poor family functioning (0-9)  *Maternal education 1*  *Maternal education 2*  *Maternal education 3* | Total- 282  98  83  101 | 30(30.6)  17(20.5)  30(29.7) | 68(69.4%)  66(79.5)  71(70.3) | 0.249 |
| Fair family functioning (10-12)  *Maternal education 1*  *Maternal education 2*  *Maternal education 3* | Total- 469  124  157  188 | 31(25.0)  44(28.0)  47(25.0) | 93 (75.0)  113(72.0)  141(75.0) | 0.780 |
| Good family functioning (13-15)  *Maternal education 1*  *Maternal education 2*  *Maternal education 3* | Total- 680  226  222  232 | 33(14.6)  45(20.3)  52(22.4) | 193(85.4)  177(79.7)  180(77.6) | 0.091 |
|  | Total 1431 |  |  |  |
| Poor family functioning (0-9)  *Maternal occupation 1*  *Maternal occupation 2*  *Maternal occupation 3* | Total- 275  58  121  96 | 12(20.7)  32(26.5)  30(31.3) | 46(79.3)  89(73.5)  66(68.7) | 0.355 |
| Fair family functioning (10-12)  *Maternal occupation 1*  *Maternal occupation 2*  *Maternal occupation 3* | Total- 449  69  200  180 | 18(26.1)  52(26.0)  48(26.7) | 51(73.9)  148(74.0)  132(73.3) | 0.988 |
| Good family functioning (13-15)  *Maternal occupation 1*  *Maternal occupation 2*  *Maternal occupation 3* | Total- 665  134  299  232 | 19(14.2)  63(21.1)  46(19.8) | 115(85.8)  236(78.9)  186(80.2) | 0.234 |
|  | Total- 1389 |  |  |  |
| Poor family functioning (0-9)    *Paternal occupation 1*  *Paternal occupation 2*  *Paternal occupation 3* | Total – 255  82  43  130 | 25(30.5)  8 (18.6)  40(30.8) | 57(69.5)  35(81.4)  90(69.2) | 0.280 |
| Fair family functioning (10-12)  *Paternal occupation 1*  *Paternal occupation 2*  *Paternal occupation 3* | Total – 434  144  71  219 | 39(27.1)  19(26.8)  54(24.7) | 105(72.9)  52(73.2)  165(75.3) | 0.858 |
| Good family functioning (13-15)  *Paternal occupation 1*  *Paternal occupation 2*  *Paternal occupation 3* | Total – 620  229  126  265 | 37(16.2)  25(19.8)  58(21.9) | 192(83.8)  101(80.2)  207(78.1) | 0.271 |
|  | Total- 1309 |  |  |  |
| Poor family functioning (0-9)  *ACEs* 0-3  *ACEs 4-5*  *ACEs 6+* | Total – 250  83  74  93 | 18(21.7)  19(25.7)  30(32.3) | ­65(78.3)  55(74.3)  63(67.7) | 0.277 |
| Fair family functioning (10-12)  *ACEs* 0-3  *ACEs 4-5*  *ACEs 6+* | Total – 426  205  113  108 | 53(25.9)  31(27.4)  25(23.2) | 152(74.1)  82(72.6)  83(76.8) | 0.761 |
| Good family functioning (13-15)  *ACEs* 0-3  *ACEs 4-5*  *ACEs 6+* | Total – 638  413  142  83 | 89(21.5)  19(13.4)  16(19.3) | 324(78.5)  123(86.6)  67(80.7) | 0.105 |
|  | Total 1314 |  |  |  |

household income 1(<1000), 2(1001-2000), 3(>2000); maternal education 1(<10), 2(10-12), 3(>12); maternal occupation 1(blue collar), 2(lower white collar), 3(upper white collar); maternal occupation1(blue collar), 2(lower white collar), 3(upper white collar); maternal age 1(13-25), 2(26-35), 3(36-47)

Supplementary Table 5. Crude association between multisite and chronic musculoskeletal pain and family functioning (overall score and dimensions: cohesion, conflict, and expressiveness)

|  | Unadjusted model (B (95% CI)) | | | |
| --- | --- | --- | --- | --- |
|  | Cohesion (0-7) | Conflict (0-6) | Expressiveness (0-2) | Family functioning score (total 0-15) |
| Multisite pain (2 and more sites) | **-0.37[-0.52, -0.23)** | **0.42[0.24,0.59]** | **-0.58[-0.11, -0.01]** | **-0.85[-1.15, -0.54]** |
| Chronic musculoskeletal pain | **-0.27[-0.44, -0.98]** | **0.31[0.10, 0.51]** | -0.04[-0.11, 0.24] | **-0.62[-0.98, -0.25]** |
